# A Wound of the Cornea Complicated by Secondary Glaucoma

**Published:** 1881-01

**Authors:** F. C. Hotz

**Affiliations:** Chicago


### ARTICLE V.

### A WOUND OF THE CORNEA COMPLICATED BY SECONDARY GLAUCOMA. By F. C. HOTZ, M.D., Chicago.

In the latter part of February, 1879, a piece of a gun-cap hit the left eye of L. H., aged fourteen years, living in a small town in Illinois. For three days the eye was very painful; after that time periods of pain alternated with free intervals, but the eye always was more or less red. On the 2d of April the boy came to Chicago. I found a great deal of photophobia, profuse secretion of tears, considerable redness around the cornea, and an irregular, ragged scar in the cornea extending obliquely from its center into the inferior temporal section. With the lower part of this scar the iris was adherent so that its tissue was forcibly dragged up toward the cornea in an abrupt fold. The adhesion was very small and did not involve the pupillary edge, the pupil, therefore, was round and mobile, and dilated readily upon the use of atropine, the portion fastened by the anterior synechia, of course, excepted. There was no trace of iritis and no disturbance of the lens; the fundus could not be sufficiently examined on account of the excessive photophobia and lachrymation. V was reduced to the counting of fingers; and T seemed increased. (T + 2). No foreign body was found in the anterior chamber; and as the piece of gun-cap pierced the cornea near the center, it could impossibly have gone beyond the anterior chamber without wounding the lens. The persistent irritation of the eye, therefore, could not be attributed to the presence of a foreign body; and as so far the eye had not received any treatment, I hoped that rest, proper care and atropine, would soon subdue the trouble.

At first, it seemed as though my expectation would be realized; the symptoms gradually subsided, and, on April 24, the eye appeared so well that I discontinued the use of atropine. No sooner was this done than the eye became again red and painful. By the re-application of atropine this relapse was subdued in two days. But now T was undoubtedly positive; pilocarpine was substituted for atropine, but was borne by the eye so ill that I returned to atropine. From this time on, the eye was very changeable; sometimes looking quite well, sometimes very red and inflamed. T continued positive; the parenchyma of the cornea began to be hazy, and the anterior portion of the sclerotic seemed to expand under the abnormally high pressure.

Excessive tension is always an evidence of an abnormal increase of the contents of the eyeball. Whether the secretion of the intra-ocular fluids is abnormally stimulated, or the excretion (filtration) of these fluids abnormally slow, in either case the disturbance of the equilibrium between production (secretion) and consumption (excretion) will result in the accumulation in the globe of a surplus of fluid; or, in other words, the eyeball becomes overfull, tense and hard. This condition of the eye is the characteristic symptom of glaucoma, and when it occurs in the course of another ocular disease, we speak of *secondary* or *consecutive* glaucoma. In the present case the glaucomatous condition was undoubtedly due to the abnormal fixation of the iris to the cornea; and consequently the only rational remedy that would afford permanent relief was an operation for the release of the iris.

May 16, I informed the father of the patient of this opinion. He did not come to the city until May 29, but at once consented to my proposition; and on the afternoon of the same day the operation was performed. About one mm. from the external margin of the cornea a narrow-bladed iridectomy knife was thrust through the transparent cornea and pushed across the iris in such direction that its point passed over the pupillary edge of the iris just above the synechia. By a slight rotation of the blade in the plane of the iris the point of the knife was made to disappear behind the iridic fold fastened to the corneal cicatrix. By further turning the blade in the same direction this fold was caught up by the cutting edge of the knife and cut off from the cornea, when the knife was withdrawn slowly from the anterior chamber. My intention was to end the operation with the release of the synechia. I was, therefore, very careful in withdrawing the knife lest the aqueous humor should escape suddenly and cause a prolapse of iris. But the intra-ocular pressure was so great that in spite of the most cautious manipulation the aqueous humor followed after the knife with such force that it carried the pupillary edge of the iris with it. Under the circumstances I considered a reposition of the prolapsed iris impracticable; I did not even make an attempt at reduction, but at once excised so much of the iris as was necessary to guard against further prolapse or anterior synechia. The day after the operation, I found the wound closed, the anterior chamber filled with aqueous humor, but T—. And T remained negative until June 4; the eye, however, was free from pain and steadily improved. I believe, therefore, that the abnormal reduction of T was due to an easy filtration of aqueous humor through the fresh cicatrix in the cornea. June

6, T was normal; and June 13, the boy could return home. The corneal parenchyma was clear; pupil horizontally oblong; fundus visible, but its vessels greatly distorted and unsteady, owing to the irregular astigmatismus of the cornea. The globe was slightly enlarged and highly myopic (as shown by ophthalmoscope); V = $\frac{10}{200}$, not improved by glasses. At an examination which I had the good cpportunity to make on October 1, I found precisely the same state of the eye. And it was not very probable that the sight would become materially better because the opaque scar extended into the central part of the cornea, causing that well marked irregularity of its refracting surface known as irregular astigmatismus.

